# On the Mechanism(s) of Membrane Permeability Transition in Liver Mitochondria of Lamprey, *Lampetra fluviatilis L*.: Insights from Cadmium

**DOI:** 10.1155/2014/691724

**Published:** 2014-06-03

**Authors:** Elena A. Belyaeva, Larisa V. Emelyanova, Sergey M. Korotkov, Irina V. Brailovskaya, Margarita V. Savina

**Affiliations:** I.M. Sechenov Institute of Evolutionary Physiology and Biochemistry, Russian Academy of Sciences, Thorez pr. 44, Saint Petersburg 194223, Russia

## Abstract

Previously we have shown that opening of the mitochondrial permeability transition pore in its low conductance state is the case in hepatocytes of the Baltic lamprey (*Lampetra fluviatilis L*.) during reversible metabolic depression taking place in the period of its prespawning migration when the exogenous feeding is switched off. The depression is observed in the last year of the lamprey life cycle and is conditioned by reversible mitochondrial dysfunction (mitochondrial uncoupling in winter and coupling in spring). To further elucidate the mechanism(s) of induction of the mitochondrial permeability transition pore in the lamprey liver, we used Cd^2+^ and Ca^2+^ plus P_i_ as the pore inducers. We found that Ca^2+^ plus P_i_ induced the high-amplitude swelling of the isolated “winter” mitochondria both in isotonic sucrose and ammonium nitrate medium while both low and high Cd^2+^ did not produce the mitochondrial swelling in these media. Low Cd^2+^ enhanced the inhibition of basal respiration rate of the “winter” mitochondria energized by NAD-dependent substrates whereas the same concentrations of the heavy metal evoked its partial stimulation on FAD-dependent substrates. The above changes produced by Cd^2+^ or Ca^2+^ plus P_i_ in the “winter” mitochondria were only weakly (if so) sensitive to cyclosporine A (a potent pharmacological desensitizer of the nonselective pore) added alone and they were not sensitive to dithiothreitol (a dithiol reducing agent). Under monitoring of the transmembrane potential of the “spring” lamprey liver mitochondria, we revealed that Cd^2+^ produced its decrease on both types of the respiratory substrates used that was strongly hampered by cyclosporine A, and the membrane potential was partially restored by dithiothreitol. The effects of different membrane permeability modulators on the lamprey liver mitochondria function and the seasonal changes in their action are discussed.

## 1. Introduction

In autumn lamprey* Lampetra fluviatilis L.* migrates from the Gulf of Finland to the Neva River (Northwestern Russia) and switches off the exogenous feeding during all the period of its prespawning migration. After the years of investigations, it became clear that a pronounced and reversible metabolic depression (for comprehensive reviews, see [1–7]) occurs in liver of the Baltic lamprey during the winter prespawning period which is characterized by substantial decrease of tissue and cellular endogenous respiration, an oxidative phosphorylation reduction, and both ATP content and mitochondrial membrane potential drop [8–13]. Besides, one of the most important peculiarities of hepatocyte energy metabolism of the “winter” lamprey is the absence of significant contribution of glycolysis in ATP production as well as its regulation via the availability of fatty acids for oxidation, which are found to be the main respiratory substrates [9–12, 14]. In spring the energy metabolism activation is observed in the lamprey liver following the appearance of the secondary sex characters [15–17]. The sharp activation of oxidation and phosphorylation in the lamprey liver mitochondria is followed by spawning and death of the animal in the end of spring—in the beginning of summer. As suggested, a strict and genetically programmed hormonal regulation of seasonal variations in metabolic rate to preserve energy resources for spawning takes place. However, molecular mechanism(s) of the phenomenon is still elusive.

Previously using isolated mitochondria as a model, we have revealed clear-cut seasonal variations of the main bioenergetics parameters of the lamprey liver [12, 14]. The found changes indicate that the metabolic depression observed during the last winter of the lamprey's life cycle is mediated by prolonged reversible mitochondrial dysfunction. The dysfunction is shown to manifest itself in the following: (1) the very low activity of mitochondrial respiratory chain, especially of its complex I, (2) low oxidative phosphorylation, (3) decreased content of mitochondrial adenine nucleotides, (4) high level of reduced pyridine nucleotides, and (5) leaky mitochondrial membranes [12, 14]. In particular, it has been found that the “winter” lamprey liver mitochondria (LLM) passively swell in an isotonic NH_4_NO_3_ solution that indicates the increased proton permeability of the inner membranes of these mitochondria. Besides, the nonenergized LLM have been found to be permeable to K^+^ and Cl^−^ as well [12, 14, 18]. The obtained data have brought us to the assumption that the enhanced membrane permeability demonstrated by the LLM in respect to protons and monovalent cations, but not to sucrose molecules, may be due to the opening of the mitochondrial permeability transition (MPT) pore [19–25] in its low-conductance state (this type of the pore conformation, as well-known, allows small solutes, like H^+^, K^+^, Ca^2+^, and so forth, to diffuse in and out of the mitochondrial matrix). Moreover, on the isolated mitochondria we have demonstrated the closing of the pore in the presence of its classical inhibitors, such as Mg^2+^, ADP (in the presence and absence of cyclosporine A, CsA), and [ethylenebis(oxyethylenenitrilo)]tetraacetic acid (EGTA), as well as after energization of the LLM [12, 14]. Since we observed the whole range of energetic states of the mitochondria (from uncoupling in winter till coupling in spring) during the prespawning migration of the lamprey, we have supposed that in vivo the MPT pore of the animal is strictly regulated [12–14, 18, 26–29].

It should be mentioned also that in our early work [29] we have got the evidence that the LLM function is sensitive to toxic action of such heavy metal, as cadmium (Cd^2+^). Before on isolated rat liver mitochondria (RLM), that is, on the conventional model to study the MPT pore involvement, we conducted a thorough investigation on mechanism(s) of Cd^2+^ toxicity and found that Cd^2+^ induced RLM dysfunction mediated by the disturbance of mitochondrial respiratory chain (mtETC) and the opening of the nonselective pore both in its low- and high-conductance states [30–37]. In the present study a one more attempt to puzzle out the mystery of life and decease of the lamprey was made. In particular, with the aim to better understand the mechanism(s) of the MPT pore induction in the lamprey liver, we used Cd^2+^ and for comparison, Ca^2+^ plus P_i_ (in some cases also a few fatty acids) as the pore inducers and the isolated mitochondria of both “winter” and “spring” animals. The seasonal changes in action of different permeability transition modulators on the lamprey liver mitochondria function were revealed and discussed. Portion of this study was presented previously in an abstract form [38].

## 2. Materials and Methods

### 2.1. Chemicals

ADP, CsA, EGTA, dithiothreitol (DTT), bovine serum albumin (BSA), nupercaine (dibucaine), carbonyl cyanide p-trifluoromethoxyphenylhydrazone (FCCP), carbonyl cyanide 3-chlorophenylhydrazone (CCCP), rotenone, oligomycin, valinomycin (Val), succinate, glutamate, pyruvate, laurate, acetate, ascorbate, tetramethyl-p-phenylenediamine (TMPD), Tris-OH, safranine, ruthenium red (RR), CdCl_2_, CaCl_2_, MgCl_2_, H_3_PO_4_ (P_i_), and so forth were purchased from Sigma (USA) or Serva (Germany); sucrose was refined with the help of a cationite KU-2-8 column; KCl was recrystallized twice; heparin, 2, 4-dinitrophenol (DNP), KSCN, KNO_3_, NH_4_NO_3_ were of analytical grade. ^137^Cs^+^ was purchased from Isotop (Russia).

### 2.2. Isolation of Mitochondria

Lampreys (*Lampetra fluviatilis L.*) of 50–70 g were caught in estuary of the Neva River in October-November during their prespawning migration from the Baltic Sea and maintained until May in holding tanks with cooled (2–6°C) and permanently aerated water. Eight to ten adult animals, if other not indicated, were taken for each experiment. The mitochondria from liver were isolated by the method of differential centrifugation in the medium of the following composition (mM): 250 sucrose, 3 Tris-HCl (pH 7.3), and 0.5 EGTA. Mitochondrial pellet was washed twice with the medium containing 250 mM sucrose, 3 mM Tris-HCl (pH 7.3). At the first washing, BSA (1 mg/mL of medium) was introduced into the medium for removing free fatty acids. Suspending of the mitochondria was conducted usually in the medium without BSA. The protein concentration was determined by Bradford or Biuret methods with BSA as standard as before [12, 14]. The mitochondrial suspension contained 25–30 mg of protein/mL. In some cases (see explanations in the text) heparin (25 or 50 units/mL) and different concentrations of nupercaine (Nup) were added in an isolation and/or assay medium.

### 2.3. Monitoring of Cs^+^ (Analog of K^+^) Uptake by Isotope Techniques

**Figure 1:**
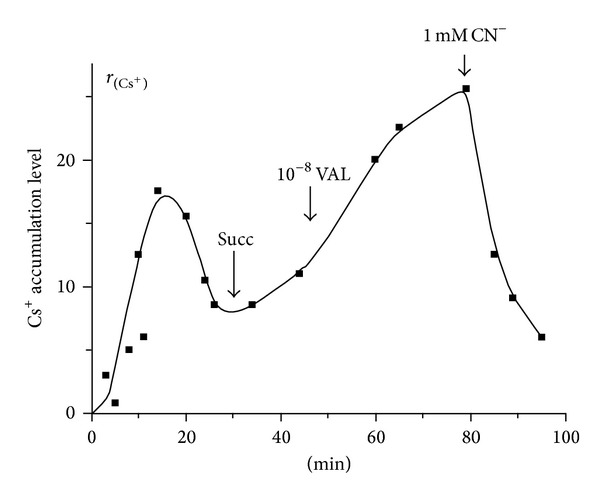
Dynamics of ^137^Cs^+^ uptake by isolated lamprey liver mitochondria (LLM) depending on energization and presence of different concentrations of valinomycin. ^137^Cs^+^ uptake by the winter LLM was measured by the Millipore filtration method as before [31, 39] in the presence of different concentrations of valinomycin in acetate medium (50 mM Tris-acetate and 140 mM sucrose). ^137^Cs^+^ accumulation level is expressed as values of coefficients of distribution (*r*_*t*_) of the corresponding tracer between the energized mitochondria and the medium (for details, see Section 2). Mitochondria (0.5 mg/mL of protein), ^137^Cs^+^ (1 *μ*M), and valinomycin (1 nM) were administered from the start of the experiment; the other additions of valinomycin (if so) were indicated by arrow. Succ (2.5 mM) was added where indicated by arrow or was presented from the beginning of measurement. [CN], if added, was 1 mM. Typical traces for four independent mitochondrial preparations are shown.

**Figure 2:**
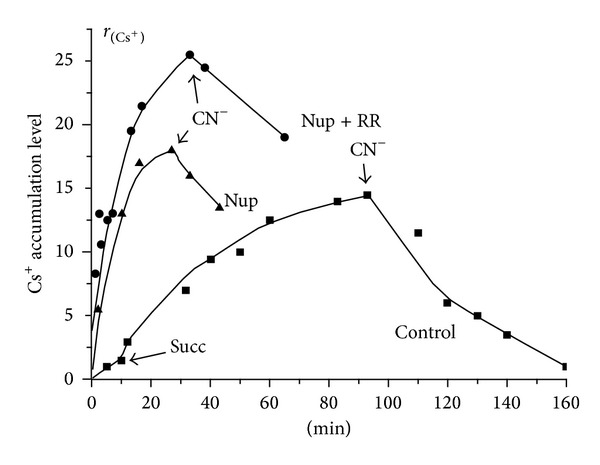
Effects of nupercaine and RR on the distribution of  ^137^Cs^+^ between isolated succinate-energized lamprey liver mitochondria and an incubation medium in the presence of valinomycin. 0.01 *μ*M of valinomycin was added from the beginning of the experiment. [Nup], [RR], and [CN], if added, were 50 *μ*M, 7 *μ*M, or 1 mM, respectively. All other details are in Figure 1.

^137^Cs^+^ accumulation by isolated LLM was determined by the Millipore filtration method, the details of which were described earlier [31, 39]. In particular, samples of 0.1 mL were taken at intervals and mixed with ice-cold medium free of the isotope, and the mitochondria were immediately filtered through filters of 0.45 mM pore size, washed once with 2 mL ice-cold medium, and assayed for their activity, with correction of filter backgrounds obtained by filtration of mitochondria-free incubation medium and washing. The uptake experiment results were expressed as the distribution ratio of the tracer (*r*_*t*_) between matrix and medium. As mentioned elsewhere [31, 39], the kinetics of the process of uptake of ^137^Cs^+^ by valinomycin-treated and substrate-energized mitochondria is well characterized by coefficients of distribution (*r*_*t*_) of the tracer between energized mitochondria and medium. These coefficients are calculated from the following equation: *r* = *A*_mito_/*A*_medium_, where *A*_mito_ is radioactivity in 1 g of mitochondrial protein and *A*_medium_ is radioactivity in 1 mL of the medium. Efflux of ^137^Cs^+^ from isolated mitochondria was estimated by the time (*t*_1/2_) required for the coefficients (*r*_*t*_) to decrease twice compared to those in the steady state. As known, the uptake of ^137^Cs^+^ by energized mitochondria in the presence of valinomycin (estimated by the coefficient *r*_*t*_) characterizes the energetic condition of isolated mitochondria and indirectly the mitochondrial membrane potential (ΔΨ_mito_) value. Incubations were carried out at 0°C, if not otherwise noted, in a medium containing 150 mM sucrose, 50 mM Tris-acetate (pH 7.4), 5 *μ*M of rotenone, 1 *μ*g/mL of oligomycin, and 0.01 *μ*M of valinomycin. Mitochondria (0.5 mg protein/mL) were energized, if not otherwise noted, by 2.5 mM Succ. For more details, see the legends to Figures 1 and 2.

### 2.4. Mitochondrial Swelling

Mitochondrial volume changes were measured from the absorbance changes at 540 nm with an appropriate recording spectrophotometer as described before [12]. In particular, reactions were carried out at 20°C in a 1.5 mL chamber filled with incubation medium, which contained 0.5 mg of mitochondrial protein. LLM membrane permeabilization was determined from the mitochondrial swelling in media containing 250 mM sucrose (or 125 mM NH_4_NO_3_ or KSCN or KNO_3_ or KCl) and 5 mM Tris-HCl (pH 7.3). The swelling of energized mitochondria in an isotonic sucrose medium is considered to be a marker of MPT pore involvement in mitochondrial membrane permeabilization [20]. It is known also that NH_4_^+^ ions are capable of crossing the membrane only as protons and freely diffusing molecules of NH_3_. The nitrate anion (NO_3_^−^) or thiocyanate anion (SCN^−^) is also freely permeable through the inner mitochondrial membrane, so the monitoring of mitochondrial swelling in NH_4_NO_3_, KNO_3_, or KSCN medium makes it possible to estimate the proton or K^+^ membrane permeability of the mitochondria, respectively. Besides, the inner membrane permeability to K^+^ was measured by monitoring the swelling of both nonrespiring and energized mitochondria in K^+^-acetate-plus-sucrose-contained media, the content of which was specified in the legends to the corresponding figures. It should be reminded that nondissociated molecules of acetic acid easily penetrate into the matrix compartment where they dissociate to acetate anions and protons. In some cases (indicated in the text) 1 *μ*M valinomycin was added to the K^+^-containing media. Besides, succinate (5 mM), rotenone (5 *μ*M), pyruvate (5 mM), malate (1 mM), oligomycin (5 *μ*g/mL), and the corresponding modulators of mitochondrial permeability transition such as Cd^2+^, Ca^2+^, P_i_, laurate, CsA, EGTA, DTT, nupercaine, and heparin were administrated into the medium as well. For the composition of media and experimental details, see the figure legends.

### 2.5. Mitochondrial Respiration and Phosphorylation

The rates of LLM respiration (oxygen consumption rates) were monitored polarographically at 25°C with a Clark oxygen electrode in a thermostatic closed chamber of 1.5 mL with magnetic stirring as before [12]. The mitochondria (1 mg of protein/mL) were added to a medium containing 250 mM sucrose, 3 mM MgCl_2_, 3 mM KH_2_PO_4_, and 3 mM Tris-HCl (pH 7.3). In some cases the respiratory buffer contained 100 mM sucrose, 40 mM KCl, 3 mM MgCl_2_, 3 mM KH_2_PO_4_, and 20 mM Tris-HCl, pH 7.3 (±50 *μ*M of EGTA and/or 1 mg/mL BSA). Besides, 5 mM pyruvate and 1 mM malate or 5 mM succinate (±5 *μ*M of rotenone) were added to the incubation medium for energization of the mitochondria; in a few sets of experiments 5 mM glutamate and 5 mM malate were used as respiratory substrates as well. There were studied various metabolic states of LLM: basal respiration, in the presence of substrates,* state 3* respiration, in the presence of substrates and ADP,* state 4* respiration, after exhausting of exogenous ADP, and uncoupled respiration, in the presence of 2, 4-DNP, FCCP, or CCCP (i.e., the maximal rate of respiration, limited only by capacity of the mitochondrial respiratory chain,* state 3u* [40, 41]). In some cases, oligomycin (1 or 5 *μ*g/mL) was supplemented in the assay medium. For further details of the experimental procedure, see text and legends to the corresponding figures. Oxygen consumption rate values are presented in nmol O_2_/min/mg of mitochondrial protein (in some cases, in ngatom O/min/mg of mitochondrial protein). Respiration control ratio (RCR,* state 3*/*state 4* or* state 3u*/*state 4*), ADP/O, and phosphorylation rate were calculated from polarographic curves.

### 2.6. Assessment of Mitochondrial Respiratory Chain Complexes Activities

The measurement of the specific activity of the individual complexes of the LLM respiratory chain was performed spectrophotometrically as described by Barrientos [42] with modifications. Activities were expressed in units per milligram of mitochondrial protein. LLM were freeze-thawed three times to obtain maximum activities.

NADH-Ubiquinone Oxidoreductase (Complex I) activity was assayed as a decrease in absorbance at 340 nm by following the oxidation of NADH. Mitochondria (40 *μ*g of protein/mL) were incubated for 2 min in 800 *μ*L of Millipore water. 200 *μ*L of reaction medium (50 mM Tris (pH 8.0), 5 mg/mL BSA, 4 mM Na azide, 4 *μ*M antimycin A, and 80 *μ*M 2,3-dimethoxy-5-methyl-6-n-decyl-1,4-benzoquinone, DB) was added and the reaction was initiated by the addition of 0.8 mM NADH. The activity of Complex I was determined using the rotenone-sensitive rate. The enzymatic activity was quantified using an extinction coefficient of 6.22 mM^-1 ^cm^−1^.

For determination of NADH-Cytochrome c Oxidoreductase (Complex I–III) activity the assay was performed at 340 nm following the increase in absorbance resulting from reduction of cytochrome c. At first, LLM were incubated in Millipore water (like for the Complex I activity determination; see above). The reaction medium containing 50 mM Tris and 5 mg/mL BSA was supplemented with 40 *μ*M oxidized cytochrome c and 4 mM Na azide. The specific rotenone-sensitive rate was calculated with an extinction coefficient of 6.22 mM^-1 ^cm^−1^.

Succinate-Ubiquinone Oxidoreductase (Complex II) activity was determined by following the secondary reduction of 2,6-dichlorphenolindophenol (DCPIP) by DB at 600 nm. The reaction mixture contained 10 mM KH_2_PO_4_ (pH 7.8), 2 mM EDTA, and 1 mg/mL BSA and 80 *μ*M DCPIP, 4 *μ*M rotenone, 4 *μ*M antimycin A, 0.2 mM ATP, 10 mM succinate, and 40 *μ*g of mitochondrial protein. The reaction was initiated by the addition of 80 *μ*M DB. The activity was expressed by using 1 mM thenoyltrifluoroacetone (TTFA).

Measurement of Succinate-Cytochrome c Oxidoreductase (Complex II-III activity) is performed at 550 nm following the increase in absorbance resulting from reduction of cytochrome c. The reaction medium contained 10 mM KH_2_PO_4_ (pH 7.8), 2 mM EDTA, 1 mg/mL BSA, 4 *μ*M rotenone, 4 mM Na azide, 0.2 mM ATP, 10 mM succinate, and the mitochondria (40 *μ*g of protein). The reaction was started by addition of 40 *μ*M oxidized cytochrome c. The activity of Complex II was measured by using antimycin A-sensitive rate.

The activity of Ubiquinol-Cytochrome c Oxidoreductase (Complex III) was assayed at 550 nm by monitoring the rate of reduction of cytochrome c by reduced DB. The reaction was initiated by addition of oxidized cytochrome c in the buffer containing 10 mM KH_2_PO_4_ (pH 7.8), 2 mM EDTA, 1 mg/mL BSA, 80 *μ*M DBH_2_, 4 *μ*M rotenone, 4 mM Na azide, 0.2 mM ATP, and the mitochondria (40 *μ*g of protein). The specific activity of Complex III was calculated by subtracting the nonenzymatic rate.

The activity of Cytochrome c-O_2_ Oxidoreductase (Complex IV) was measured by following the oxidation of reduced cytochrome c as a decrease in absorbance at 550 nm. The reaction buffer contained 50 mM H_2_PO_4_ (pH 7.0), 1 mg/mL BSA, 0.2% Tween, and the mitochondria (40 *μ*g of protein). The reaction was started by adding 20 *μ*M of reduced cytochrome c.

The activities of Complexes II, III, IV, I–III, and II-III were calculated by using 19.1 mM^-1 ^cm^−1^ extinction coefficient.

In addition, quantitative determination of cytochromes in the LLM was also performed using the method of differential spectrophotometry as before [14]. In particular, for oxidation-reduction of the cytochromes medium of incubation and corresponding additions analogous to those described in [43] was used. The recording of spectra was performed on a Specord M-40 spectrophotometer (Germany) and the concentrations of cytochromes c, c1, and aa3 were calculated according to [43].

### 2.7. Determination of Mitochondrial Membrane Potential (ΔΨ_mito_)

An estimation of LLM membrane potential (ΔΨ_mito_) was conducted according to [44], with the help of fluorescent lipophilic cation-safranine O, using a Shimadzu RF-1501 spectrofluorometer (Japan) at the excitation wavelength of 485 nm and emission wavelength of 590 nm as before [27, 28]. The dynamics of ΔΨ_mito_ dissipation was determined also by the Millipore filtration method (see Section 2.3). For more details see legends to Figures 1, 2, and 6.

### 2.8. Data Analysis

Data were expressed as mean values ±SE. The results shown are mean or representative of a series of at least three independent experiments, unless otherwise indicated. The statistics were analyzed with ANOVA and Student's *t*-tests, with *P* < 0.05 assumed as the significance threshold. The mitochondrial respiration and swelling curves were plotted with the help of the program Origin 6.0.

## 3. Results and Discussion

### 3.1. Seasonal Changes in Action of Different Membrane Permeability Modulators on the LLM Function

With the aim to obtain more tightly coupled mitochondria from lamprey tissues, namely, from lamprey liver, for working during a whole year not depending on a season, in our early works [8, 9, 18] we modified isolation and incubation conditions; in particular, we found that in December–February LLM with the highest RCR (see Section 2.5) could be obtained when the isolation and/or incubation medium was supplemented by nupercaine, Nup (also known under the name of dibucaine), and/or heparin and/or BSA. Contrary, in our late works [12, 14] with the aid to study the season changes in bioenergetics parameters of LLM, we removed the above mentioned substances from the isolation/incubation procedure. To better understand the mechanism(s) of the improving action of these compounds, in this work we investigated more thoroughly the effects of these reagents on the in vitro mitochondrial function of the lamprey liver.

Using the Millipore filtration method (see Section 2.3 and [31, 39]), we studied an uptake of ^137^Cs^+^ by isolated winter LLM depending on energization in the absence and in the presence of different concentrations of Val in acetate medium with or without Nup and/or RR (Figures 1 and 2). As known, the uptake of ^137^Cs^+^ by energized mitochondria in the presence of Val (estimated by the coefficient *r*_*t*_) characterizes the energetic condition of isolated mitochondria and indirectly the ΔΨ_mito_ value. This method of ΔΨ_mito_ monitoring is especially convenient for determination of the dynamics of ΔΨ_mito_ dissipation. We found herein (see also [18, 45]) that at 0°C the nonenergized winter LLM were practically not able to accumulate ^137^Cs^+^ in the absence of Val in the assay medium. As seen from Figure 1, an addition of 10^−9^ M of Val into the medium resulted in the significant ^137^Cs^+^ uptake by the mitochondria, which further was followed by decrease of the distribution ratio of ^137^Cs^+^ (*r*_Cs_) between matrix and medium due to the developing diffusion potential dissipated with time. An energization of the mitochondria by Succ and especially an additional supplement of Val (10^−8^ M) into the medium produced the sharp rise of *r*_Cs_ that, in turn, was declined by addition of 1 mM NaCN (Figure 1). As seen from Figure 2, an addition of Nup (50 *μ*M) into the assay medium increased the rate of ^137^Cs^+^ uptake by winter LLM, and the simultaneous addition of RR (7 *μ*M) still more enhanced this effect. It should be stressed that the values of *r*_Cs_ found herein for winter LLM were 15–20 times lower than those of RLM measured by us before under the same conditions [30–34]. The lower rates of ^137^Cs^+^ uptake by the LLM and the lower values of *r*_Cs_ (compared with RLM) are in a good accordance with the increased ion membrane permeability of winter LLM found in our previous works [12, 14]. A one more marked distinction we have found between winter LLM and RLM relates to the effect of Nup on the functioning of these mitochondria. Previously on isolated RLM [30–34] we showed that Nup (i.e., local anesthetics and an amphiphilic cation which is known to affect membrane lipid-protein interactions and is considered to be effective MPT pore inhibitor [46]) did not improve the function of untreated (control) RLM that is in contrast to the situation found herein for the winter LLM the energetic condition of which became better after addition of the effector under test (see Figure 2). Moreover, as we revealed earlier [33], Nup per se (taken in concentration of 50 *μ*M) produced significant decrease in ^137^Cs^+^ uptake level of Succ-energized RLM incubated in isotonic (sucrose plus acetate)-contained medium supplemented by Val, oligomycin, and rotenone, that is, under the same experimental conditions which are used in this study and presented on Figure 2. Likewise, we have found that Nup does not improve the functioning of the spring LLM and sometimes even made them worse (data not shown).

Practically the same situation we observed under studying the action of heparin on the in vitro LLM function herein and before [12, 14]. As known, heparin is a sulfated anionic glycosaminoglycan widely used as an anticoagulant in clinical practice for injections and to form an inner anticoagulant surface on various experimental and medical devices such as test tubes and renal dialysis machines [47, 48]. Heparin and heparin-like glycosaminoglycans can modulate the cationic environment in different physiological systems, interacting with such vital metal cations as iron, copper, and calcium [49–52]. It is known also that heparin suppresses lipid raft-mediated signaling in various biological systems [53]. In our hands, the mitochondrial bioenergetics parameters of the winter lamprey liver such as basal respiration rate, ADP-stimulated (phosphorylating) respiration rate, and RCR (see Section 2.5) were found to be significantly affected by heparin and some MPT inhibitors (ADP, Mg^2+^, CsA, EGTA, BSA, RR, etc.); namely, they were improved in some extent by supplement of these reagents, while an influence of these chemicals on the uncoupler-stimulated mitochondrial respiration rate (the maximal respiratory rate, limited only by capacity of the mitochondrial respiratory chain [40, 41]) depressed strongly during synchonia was marginal. Besides, heparin added to the isolation and/or incubation medium decreased partially the enhanced ion permeability of the nonenergized winter LLM (see also [12, 14]). Opposite to this, heparin did not produce any improving action on the function of the spring LLM.

In this connection, it should be reminded about the very interesting data which was found by one of us more than 30 years ago, concerning the action of heparin on the mitochondrial reverse electron transport in the lamprey liver (i.e., from succinate to NAD^+^ via Complex I of the mitochondrial respiratory chain) [9]. In particular, Savina and Derkachev revealed that in the winter LLM the reverse electron transport which registered through changes in the pyridine nucleotide fluorescence according to [54] was disturbed. Remarkably, in the winter LLM isolated in the presence of heparin (50 units/mL) the reverse electron transport was completely restored. The occurrence of the mitochondrial reverse electron transport in the LLM was used by us as an additional test for coupling of the mitochondria. It was suggested that the unknown factor, which uncoupled LLM and was opposed by heparin, existed only at lower temperatures (4-5°C) and vanished after adaptation of winter lampreys to elevating temperatures (from 1 to 18 days at 17°C), because the liver mitochondria of the adapted winter lamprey were tightly coupled, including the restored reverse electron transport via Complex I. Moreover, these mitochondria resembled strongly liver mitochondria of mammals, such as rat and mouse. Importantly, after placing the adapted lampreys to cold water, the mitochondria again became uncoupled that points to the existence of a low-temperature hibernation period in the lamprey's life cycle during synchonia [9].

Thus, all this agree well with our recent findings pointing to the opening of the MPT pore in its low-conductance state in the winter LLM as well as with the suggestion about possible involvement of Complex I of the mitochondrial respiratory chain in this process ([12, 14], see also [55] and references therein). It should be stressed also that in our further experiments, the results of which are presented in this work, we used both heparin-treated and nontreated LLM depending on the task and the season.

### 3.2. Seasonal Changes in Respiratory Chain Complexes Activities in the LLM

In our previous works ([12, 14], see also Figure 5 herein) we have shown that the respiration rates of the LLM in various metabolic states (see Section 2.5) display strong seasonal differences depending on substrate used for oxidation. In particular, in winter (December–February) the rates of oxidation of NAD-dependent substrates, pyruvate, and malate (i.e., Complex I substrates) by the LLM were depressed significantly in all metabolic states under study while in spring (April) the rates of oxidation of the NAD-dependent substrates in* state 4* increased 3 times, in the* state 3*, 6 times and in the* state 3u*, 7-8 times; besides, the respiratory controls increased 2 times and the rates of phosphorylation, 7–10 times. At the same time, the rates of oxidation of FAD-dependent substrates, namely, succinate (i.e., Complex II substrates), both in the presence and in the absence of rotenone (Complex I inhibitor) did not have such pronounced seasonal changes. In spring, their values increased 1.3–2 times (depending on the metabolic state of the mitochondria) as compared to that in winter.

As well-known, three mitochondrial respiratory chain components are involved in oxidation of NAD-dependent substrates, such as pyruvate, malate, and glutamate, namely, Complex I (EC 1.6.99.3), Complex III (EC 1.6.5.3), and Complex IV (EC 1.9.3.1). In oxidation of FAD-dependent substrates, such as succinate, Complex II (EC 1.3.99.1) participate in combination with Complexes III and IV. We have found that the content of cytochromes c, c1, and aa3 does not change throughout the whole prespawning period (November–May): 0.15–0.17, 0.11–0.13, and 0.19–0.21 nmol/mg of mitochondrial protein, respectively. We proposed that the same was likely true for Complex II, as the rate of succinate oxidation in the presence of an uncoupler (i.e., the maximal rate of respiration, limited only by capacity of the mitochondrial respiratory chain,* state 3u* [40, 41]), did not differ significantly during the indicated seasons (e.g., in the case of 2, 4-DNP it was 18.7 ± 0.2 nmol O_2_/min*·*mg protein in winter and 25.1 ± 6.0 nmol O_2_/min*·*mg protein in spring). We suggested also that Complexes III and IV remained equally active at the winter and spring periods of prespawning migration. In contrast, the rates of oxidation of NAD-dependent substrates (pyruvate and malate) in the presence of 2, 4-DNP substantially decreased at the winter period (4.4 ± 0.6 nmol O_2_/min*·*mg protein) as compared with spring (31.4 ± 8.3 nmol O_2_/min*·*mg protein). Since the activity of Complexes III and IV participating in the NAD-dependent substrates oxidation did not change during the prespawning period, one had to accept that during winter the respiratory Complex I was inactivated. Importantly, the data obtained with heparin and under using of the thermal adaptation (see Section 3.1 and [9]) also indicated that some substantial and reversible changes in functioning of the LLM respiratory Complex I (in its activity, and/or in its conformation, and/or in its redox regulation) took place during the prespawning period.

**Figure 3:**
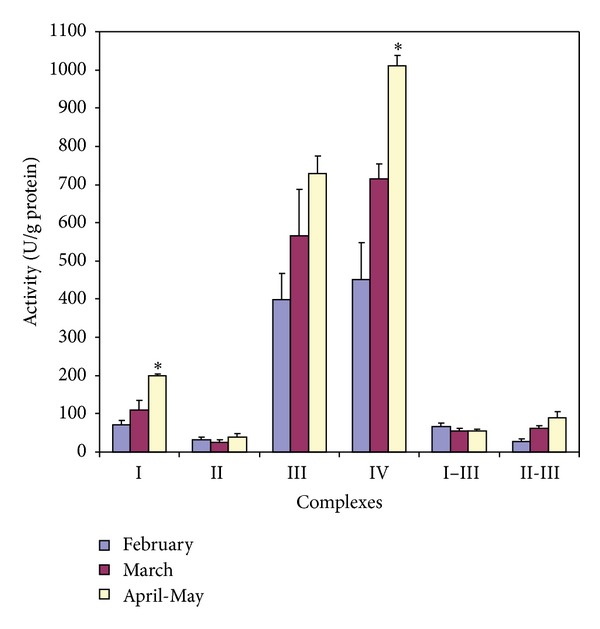
Mitochondrial respiratory chain complexes activities in lamprey liver mitochondria depending on season. Enzymatic activities were determined as described in Section 2 and expressed in terms of nmol/min/mg protein. Data were analyzed by ANOVA using 6.0 statistical software. Means ± SE are plotted (*n* = 5). *—significantly different from the winter value (at *P* < 0.05).

In the present study we decided to measure seasonal changes (if so) in respiratory chain complexes activities in the LLM. In Figure 3 the activities of respiratory chain complexes in LLM in different periods of synchonia are demonstrated (in units/g protein). It is seen that there are no significant seasonal differences in Complex II, Complex III, Complex I–III, and Complex II-III activities. Complex I activity increased in March (110.5 ± 24.5) and April (199.1 ± 6.0), as compared with that in February (71.9 ± 9.9). Complex IV activity increased more than in one and a half—two times in spring (from 451.7 ± 95.1 in February to 714.1 ± 40.4 and 1011.2 ± 27.2 in March and April, resp.). The differences between the activities in February and in April were significant for both Complex I and Complex IV (Figure 3).

The shown data, namely, the substantially decreased activity of Complex I and the nonchanged activity of Complex II in the winter LLM (see Figure 3), agree well with the data obtained from respiratory experiments conducted by us before [12, 14] and herein; in particular, as seen from Figure 5(a), in winter the FAD-linked LLM respiration (trace 5) is only partially suppressed in contrast to the NAD-linked one, which is practically switched off (trace 4). This was especially evident, when the mitochondria were isolated in the absence of heparin (data not shown). Notably, in spring the LLM respire well on both types of respiratory substrates used not depending on the presence of heparin, BSA, and so forth in the isolation/incubation medium (Figure 5(c)).

In this connection, it should be stressed that in different types of cells mitochondrial respiratory Complex I is found to be a subject of reversible covalent regulation by phosphorylation and dephosphorylation [56, 57] and by thiol-based redox modifications (for the recent comprehensive reviews, see [58, 59]). It seems quite possible that something like that may relate to mechanism(s) of seasonal regulation of Complex I in the lamprey liver as well.

In particular, it is known now that control of the catalytic activity of mitochondrial Complex I by the so-called active/deactive (A/D) transition involves a specific and well defined cysteine switch mechanism [58–60]. The phenomenon of the A/D transition was revealed for the first time by Kotlyar and Vinogradov in submitochondrial particles from bovine heart [61], and later it was found in intact mitochondria as well [62]. The A-form (i.e., active) of the enzyme catalyzed rotenone-sensitive NADH-ubiquinone reductase reaction, whilst the D-form (i.e., deactive) could be fully reduced by NADH and oxidized by artificial electron acceptors but was unable to transfer electrons to ubiquinone. An equilibrium between the A- and the D-forms of the enzyme (greatly shifted to the latter one) was reached in the absence of any ligands and the rate of equilibrium was exceptionally temperature-dependent (negligible at 15–20°C but significant at 30–37°C). Markedly, rotenone influenced on the spontaneously established equilibrium, namely, it partially protected and partially reversed the thermally induced deactivation; moreover, rotenone had almost two order of magnitude higher affinity for the A-form. In addition, only the thermally deactivated enzyme was irreversibly inhibited by SH-reagents such as N-ethylmaleimide. There were some other important differences between these two forms. In particular, the D-form was strongly inhibited by Ca^2+^ while there was no sensitivity of the A-form to this cation. The affinity of the D-form to polyvalent cations was found to be in the following order: Ni^2+^ > Co^2+^ > La^2+^ > Mn^2+^ > Ca^2+^ = Mg^2+^ > Ba^2+^; besides, its affinity to Ca^2+^ was strongly pH-dependent. Furthermore, Ca^2+^ increased the reactivity of the enzyme sulfhydryl group in the D-form towards N-ethylmaleimide. It was shown also that divalent cations like Ca^2+^ [63] and covalent modification of the single cysteine [64] can prevent reactivation of deactive Complex I. Importantly, because the divalent metal binding site and the cysteine are only accessible in the D-form of the enzyme, this makes the A-form resistant to this type of inhibition [60–64]. In addition, as found later by Maklashina et al., the A/D transition is an attribute of Complex I from vertebrates [65]. Interestingly, in the lower eukaryotes A/D transition of Complex I is also observed, but deactivation is faster and takes place at lower temperatures. Notably, during the last decade the evidence is accumulated, indicating that the A/D transition is more than an in vitro phenomenon [66–69]. In particular, Maklashina et al. revealed its functional connection with anoxia-reperfusion injury, using Langendorff hearts [66]. Galkin and Moncada have shown induction of the D-form of Complex I during prolonged hypoxia and have suggested that this could make the cysteine exposed only in this state accessible for nitrosation that finally could result in permanent deactivation of the enzyme and implicate for ischemic injury [67, 68]. Besides, Prime et al. recently have found significant cardioprotective effects in mice associated with the S-nitrosation of the cysteine switch of the A/D transition [69]. All these results demonstrate that the A/D transition and the associated cysteine switch are operational in vivo.

The same is possible to say for the situation observed in hepatocytes of the winter lamprey. As mentioned above, Complex I is inactivated spontaneously (converts into the D-form) in the absence of substrates. As found, the fatty acids (FA) serve the main energy substrate for the lamprey liver mitochondria during winter and spring prespawning periods. NADH generated in the process of *β*-oxidation of FA is then oxidized by the respiratory chain Complex I. It seems that suppression of the energy metabolism in the lamprey liver cells in winter starts from an inactivation of the Complex I of the mitochondrial respiratory chain, one of the causes of which, the most likely, is inaccessibility at this period of FA—the main energy substrate for the hepatocytes. It is known also that the D-form of Complex I slowly turns back into the A-form in the presence of NADH (or NADPH) and ubiquinone. So, in spring the transition of Complex I from inactive to active forms likely to occur, when under effect of estrogen the swift lipolysis of fats stored in the hepatocytes in the form of lipid droplets begins and their energy substrates, FA start coming to the mitochondria [12, 14]. Besides, the data concerning the action of heparin and the thermal adaptation on the mitochondrial reverse electron transport in the lamprey liver strongly support the idea that mechanism(s) of seasonal regulation of Complex I in the lamprey liver can involve the A/D transition of the complex. Moreover, the transition from one form to another is associated with the conformational alterations of the enzymatic complex by means of which, in opinion of several authors including us, the mitochondrial respiratory chain components (individual complexes and/or supercomplexes) can participate in regulation (and even in formation) of the Ca^2+^-dependent nonselective pore ([23, 25, 33, 55, 70] and references therein).

### 3.3. Effects of Cd^2+^, Ca^2+^, *P*_*i*_, and Fatty Acids on the LLM Function and Their Season Dependence

#### 3.3.1. Action on the LLM Membrane Permeability

As mentioned above, in our previous works [12, 14] we have found that the reversible mitochondrial dysfunction (mitochondrial uncoupling in winter and coupling in spring) is observed in the lamprey liver during the metabolic depression in the last year of the lamprey life cycle. Importantly, the LLM dysfunction is found to manifest itself in the significant mitochondrial membrane permeabilization. It should be reminded, however, that the winter LLM are highly permeable only to different ions, including protons, but not permeable to sucrose. Besides, proton membrane permeability of the LLM depends on season and energization. In particular, the winter LLM are highly permeable to protons and swell extensively in 125 mM NH_4_NO_3_ medium both in the absence and in the presence of the oxidized substrates. In winter the passive swelling intensity in NH_4_NO_3_ medium is about 30% higher than that in April, and the permeabilization is completely insensitive to CsA. As reported elsewhere, CsA (i.e., a potent pharmacological desensitizer of the classical nonselective pore in the inner mitochondrial membrane) is incapable to close the pore in mitochondria depleted by ATP and ADP [20–25]. Before we have found that the content of ATP and ADP in the LLM, which is low per se, decreases in winter by more than 20–30% [10, 11], so we have speculated that the loss of matrix adenine nucleotides could be one of possible explanations why CsA does not have any effect on the winter nonenergized mitochondria. In spring the passive proton permeability of the LLM membrane becomes lower, while the intramitochondrial levels of ATP and ADP get higher. As a result, the inhibitory (although moderate) influence of CsA on the passive mitochondrial swelling in NH_4_NO_3_ medium is observed. As to the energized LLM, we have found that they are highly permeable to protons in winter whilst in spring they lose this capability. Besides, the energized winter mitochondria are susceptible, although in a different extent, to several MPT pore inhibitors taken separately or in combination, such as Mg^2+^, ADP, EGTA, RR, BSA, heparin, nupercaine, and CsA. However, both nonenergized and energized winter LLM were not sensitive to a dithiol reducing agent, dithiothreitol, DTT ([12, 14] and herein).

**Figure 4:**
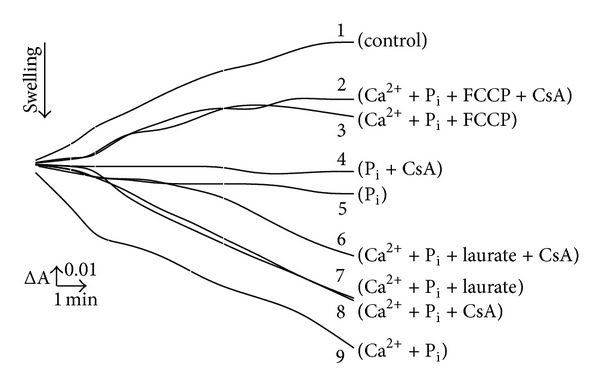
Action of Ca^2+^, P_i_, laurate, and FCCP on the membrane permeability of winter lamprey liver mitochondria. A membrane permeabilization of isolated lamprey mitochondria was determined from their swelling (measured spectrophotometrically as apparent absorbance changes at 540 nm) in 250 mM sucrose medium (3 mM Tris-HCl, pH 7.3) at 20°C in the presence of succinate, rotenone, and oligomycin; mitochondrial protein content, 0.5 mg/mL. The concentrations used are: Ca^2+^, 20 *μ*M; P_i_, 1 mM; FCCP, 100 nM; laurate, 30 *μ*M; CsA, 2 *μ*M. Experiments were repeated five times, with data presented being representative.

To better understand the mechanism(s) of induction of the MPT pore in the lamprey liver and especially its apparent seasonal dependence and physiological relevance, in the present work we continued studies in this direction. For this purpose, we used heavy metal ion Cd^2+^ (a potent dithiol reagent and Ca^2+^ agonist) and for comparison, Ca^2+^ plus P_i_ (in some cases also several fatty acids, mainly laurate) as potential MPT pore inducers and the isolated mitochondria of both “winter” and “spring” animals. We found that Ca^2+^ plus P_i_ induced the high-amplitude swelling of the isolated winter LLM both in isotonic sucrose and ammonium nitrate medium while both low and high Cd^2+^ (1–10 *μ*M and up to 1 mM, resp.) per se did not produce the mitochondrial swelling in these media. In particular, as seen from Figure 4, Ca^2+^ (20 *μ*M), P_i_ (1 mM), and laurate (30 *μ*M) applied separately or in combination produce high-amplitude swelling of the winter LLM energized by succinate (Succ) in the sucrose medium and it is only partially sensitive to CsA. Moreover, both natural (laurate) and artificial (p-trifluoromethyl carbonyl cyanide phenylhydrazone, FCCP) uncouplers depressed markedly the Ca^2+^ plus P_i_-promoted swelling of the lamprey mitochondria oxidizing Succ in sucrose medium (Figure 4). It seems also that the Succ-energized untreated (control) winter LLM shrink in this medium (Figure 4, trace 1). The addition of P_i_ per se to the medium (Figure 4, trace 5) stops this apparent shrinkage that correlates well with recently revealed inhibitory potency of P_i_ [25]. In opposite, Cd^2+^ did not induce any swelling of the winter LLM in the sucrose medium at all concentrations under study and all experimental conditions used (including the presence of heparin in isolation and/or incubation media). This sharply differ them from RLM where Cd^2+^ was found to be the very effective MPT inducer [30–36]. The close situation was found by us for other heavy metals, namely, for Hg^2+^ and Cu^2+^ (data not shown). Not long ago it was shown also that isolated hepatocytes of the winter lamprey were highly tolerant to exposure with different metabolic inhibitors, such as rotenone, antimycin A, and oligomycin, and their viability in the presence of these stressors are temperature- and pH-dependent [13]. All these findings are in a good accordance with data existed in literature in the field which obtained on different species, such as oysters, shrimps, birds, bats, and naked mole rats [4, 6, 7, 71–75].

In addition, we revealed the clear-cut season dependence in action of laurate on the LLM function that will be discussed elsewhere. Briefly, the action of laurate as the pore inducer depended on energetic condition of the mitochondria, namely, in winter in the low-energetic LLM laurate decreased the inducing action of Ca^2+^ plus P_i_ (see Figure 4, trace 7), whereas in spring it enhanced the action of these effectors. It is worthy of note also that in spring more than two times higher concentrations of Ca^2+^, P_i_, and laurate for the pore induction in the LLM were needed (data not shown).

#### 3.3.2. Action on the LLM Respiration

**Figure 5:**
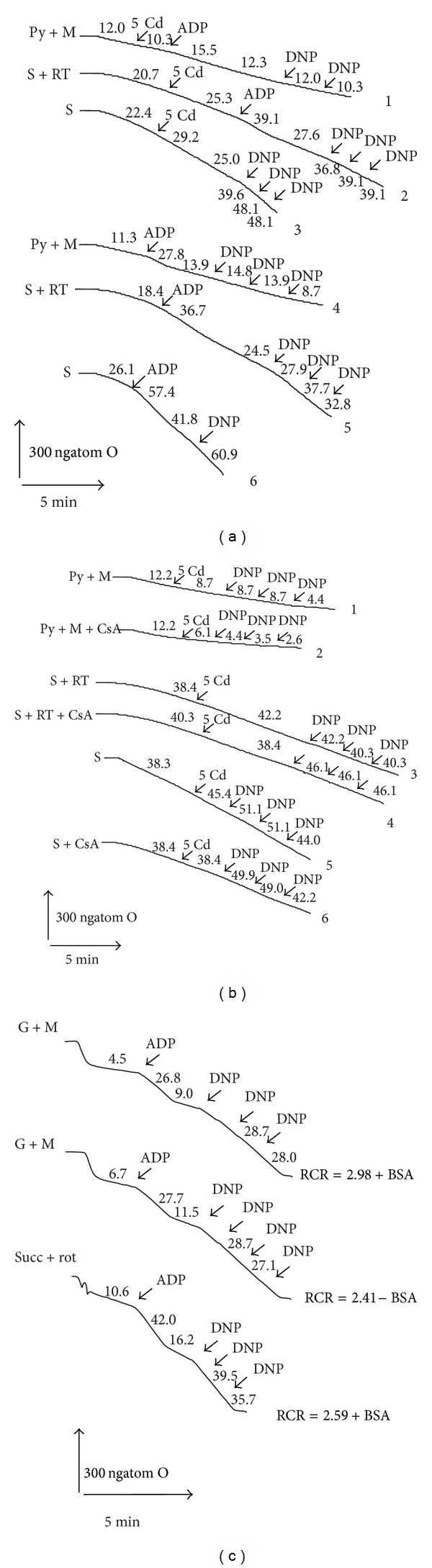
The lamprey liver mitochondrial respiration depending on the season, the substrate used and the presence of Cd^2+^ and/or cyclosporine A. The rates of LLM respiration (oxygen consumption rates) were monitored polarographically at 25°C with a Clark oxygen electrode in a thermostatic closed chamber of 1.5 mL with magnetic stirring. The mitochondria (1 mg of protein/mL) were added into a medium containing 250 mM sucrose, 3 mM MgCl_2_, 3 mM KH_2_PO_4_, 3 mM Tris-HCl, pH 7.3 ((a) and (b), winter LLM) or 100 mM sucrose, 40 mM KCl, 3 mM MgCl_2_, 3 mM KH_2_PO_4_, and 20 mM Tris-HCl, pH 7.3 (±50 *μ*M of EGTA and/or 1 mg/mL BSA) ((c), spring LLM). 5 mM pyruvate or glutamate and 1 or 5 mM malate or 5 mM succinate (±5 *μ*M of rotenone) were added to the incubation medium for energization of the mitochondria. The concentration of oligomycin, if added, was 1–5 *μ*g/mL. The basal respiration rate (in the presence of substrates), in state 3 (substrates and 50 *μ*M ADP), in state 4 (after exhausting of ADP) and uncoupled respiration rate (2, 4-DNP was added in state 4) were estimated. The respiration rates (ngatom O/min/mg of protein), respiratory control ratios [VO_2_(3)/VO_2_(4)], ADP/O_2_, and the phosphorylation rate were calculated from polarographic curves. Typical traces for three independent mitochondrial preparations are shown.

It is well known now that the respiratory function of the lamprey liver is a subject of season regulation; besides, the respiration of isolated winter lamprey mitochondria is substrate-dependent ([12, 14] and Figure 5 herein). Substantial differences in the action of different MPT pore modulators in the presence of NAD- or FAD-dependent respiratory substrates depending on season were revealed as well. In the present work we studied the effects of different concentrations of Cd^2+^ on the LLM respiration in different metabolic states in the presence and in the absence of CsA. As seen from Figure 5(a), in the winter LLM low Cd^2+^ (5 *μ*M) decreased the ADP-stimulated (phosphorylating) respiration rate (i.e., in state 3) and the DNP-uncoupled respiration rate (i.e., the maximal respiratory rate, limited only by the capacity of the mitochondrial respiratory chain [40, 41]) as well as RCR (respiratory controls) on both types of respiratory substrates used; however, all the Cd^2+^ effects were less pronounced as compared with that in RLM [30–36]. Notably, Cd^2+^ action on the basal respiration and on the resting state respiration (i.e., in state 4) was strongly dependent on the substrate used. In particular, we found that low Cd^2+^ enhanced the inhibition of the basal and state 4 respiration rates of the lamprey mitochondria oxidizing NAD-dependent substrates, namely, Glu plus Mal or Pyr plus Mal (Figure 5(a), traces 1 and 4), whereas it evoked their partial stimulation on FAD-dependent substrates, namely, on Succ in the presence (Figure 5(a), traces 2 and 5) and in the absence of rotenone (Figure 5(a), traces 3 and 6). Besides, all the aforementioned changes in the respiration rates of the winter LLM promoted by the heavy metal were only weakly (if so) sensitive to CsA (the MPT pore desensitizer) added alone and in combination with oligomycin (Figure 5(b)). The Cd^2+^ effects were revealed in the LLM isolated both in the presence and in the absence of heparin.

#### 3.3.3. Action on the LLM Membrane Potential (ΔΨ_mito_)

As we found before under measuring the dynamics of the lamprey liver mitochondrial membrane potential (during the whole prespawning migration period of the animal) estimated by accumulation of fluorescent lipophilic cations, safranine O by isolated mitochondria [27, 28] or TMRM (tetramethylrhodamine methyl ester) by isolated cells [13], the ΔΨ_mito_ changes were strongly dependent on season and types of substrates used for energization (the latter—for isolated LLM). In particular, it was found on the isolated hepatocytes that the ΔΨ_mito_ values were maximal in October and March-April, while in winter its values were substantially declined that provoked likely by prolonged starvation and profound metabolic arrest [13]. On the isolated winter LLM it was shown also that the intensity of safranine O fluorescence reflecting the membrane potential was lower at the pyruvate and malate oxidation during the period of metabolic depression and dissipated much more rapidly than at oxidation of succinate [27]. Remarkably, the addition of ADP or/and EGTA (i.e., the potent MPT effectors) into incubation medium led to the significant delay of the membrane potential dissipation of the winter LLM energized by NAD-depended substrates that strongly supported the suggestion about the involvement of the Complex I in the process of the pore formation and/or regulation.

In the present work we investigated the action of Cd^2+^ on the ΔΨ_mito_ changes of the isolated LLM energized by different types of respiratory substrates in the presence and in the absence of CsA (the strong MPT pore desensitizer) and/or DTT (the potent dithiol reductant).

**Figure 6:**
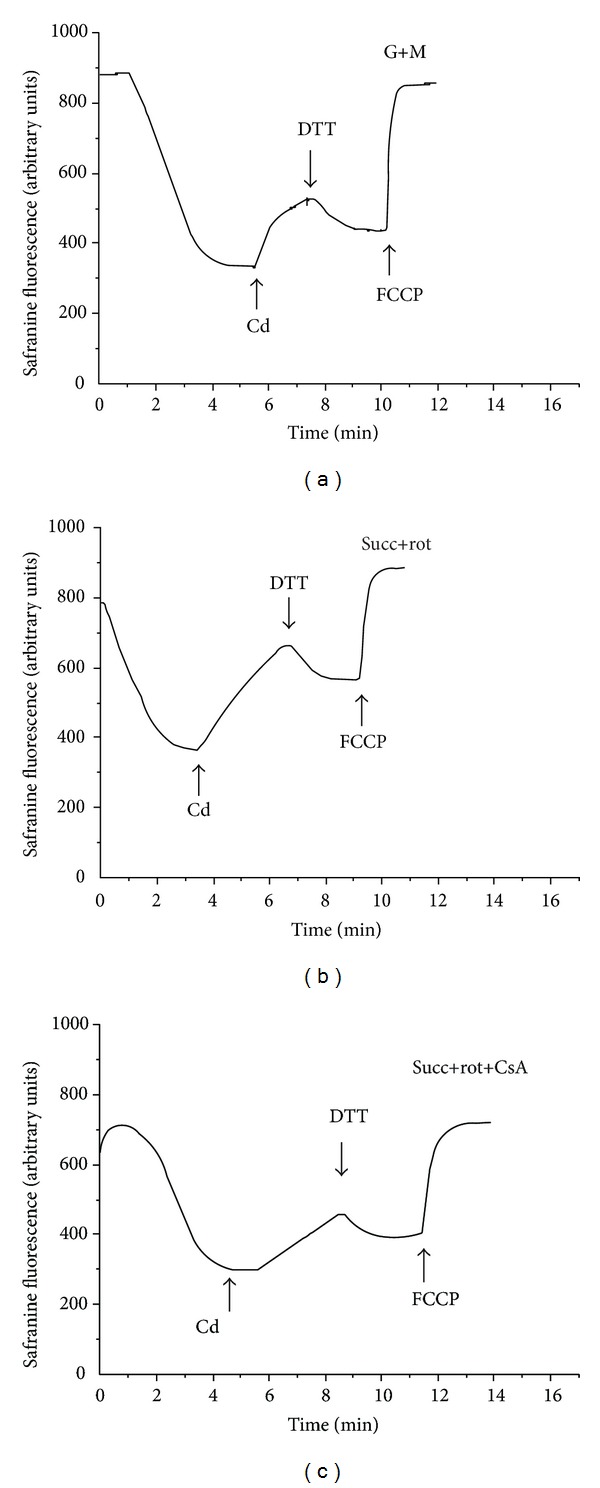
Action of Cd^2+^ on the transmembrane potential of spring lamprey liver mitochondria in the absence or presence of cyclosporine A and/or dithiothreitol. The lamprey liver mitochondrial membrane potential was estimated with the help of fluorescent lipophilic cation, safranine O, by measuring changes in intensity of its fluorescence (in arbitrary units) on a Shimadzu RF-1501 spectrofluorometer at the excitation wavelength of 485 nm and emission wavelength of 590 nm. Mitochondria (0.5 mg/mL of protein) were added into a medium containing 250 mM sucrose, 3 mM MgCl_2_, 3 mM KH_2_PO_4_, 3 mM Tris-HCl (pH 7.3), and 3 *μ*g/mL of oligomycin and 4.5 *μ*M of safranine O, as well as respiratory substrates: 5 mM of glutamate and 5 mM of malate, or 5 mM of succinate plus 5 *μ*M of rotenone. Cd^2+^ (5 *μ*M), DTT (1 mM), and FCCP (1 *μ*M) were added where indicated by arrows. [CsA], if added, was 1 *μ*M. Typical traces for 3–5 independent experiments are shown.

As seen from Figure 6, in spring the LLM were found to be highly sensitive to Cd^2+^ when energized by both NAD- and FAD-depended substrates. In particular, under monitoring of the transmembrane potential of the “spring” lamprey liver mitochondria with the help of safranine O, we revealed that Cd^2+^ produced the significant decrease of the ΔΨ_mito_ on both types of the respiratory substrates used that was strongly hampered by CsA, and the membrane potential was partially restored by DTT (Figure 6).

So, the data obtained by us herein and before indicate that in winter the pore in the LLM opened in its low-conductance state is Ca^2+^-dependent, sensitive to Mg^2+^, ADP, CsA, heparin, nupercaine, and so forth (although in a different extent) but not sensitive to DTT. Moreover, the winter LLM were highly tolerant (in some aspects) to such potent thiol reagents and inducers of oxidative stress and MPT, as heavy metals; this sharply distinct them from RLM. On the contrary, in spring the LLM resemble RLM very much, including the sensitivity to Cd^2+^ and DTT (Figure 6). These facts combined with the observations of high level of reduced PN in the LLM point to the absence of the oxidative stress in the winter lamprey hepatocytes. At present it is clear that the energetic condition of the lamprey hepatocytes is strictly regulated; however, signaling pathways involved in this regulation are so far unknown. In our opinion, one of these pathways probably connect with creation of inaccessibility of some critical thiol(s) most likely related to the A/D transition of Complex I and involved in regulation or/and formation of the MPT pore (see Section 3.2 and [58, 59, 64–69]). Importantly, the absence of the severe oxidative stress may be one of the main causes of reversibility of the metabolic depression observed in the lamprey hepatocytes during prespawning migration of the animal in contrast to the irreversible metabolism suppression and mitochondrial dysfunction revealed in various types of cells under different diseases and pathological conditions [76–78].

### 3.4. Concluding Remarks

In conclusion, it should be stressed that liver mitochondria and hepatocytes of the Baltic lamprey and the season changes observed in their functioning are an excellent “natural” model for elucidating mechanism(s) of suspected participation of the individual mitochondrial respiratory chain complexes (and/or their supercomplexes?) in formation and/or regulation of the enigmatic MPT pore (see [25, 55, 59, 70] and references therein), which at the moment is proved to play the key role in the induction of different types of cell death. In particular, the mechanism(s) of the reversible mitochondrial dysfunction observed in liver of the starving lamprey during the prespawning period of its life cycle, as mentioned above, include the interrelated reversible the season-dependent opening of the MPT pore in its low-conductance state and the disturbance of Complex I activity (including its reverse electron transport) that indicate to possible involvement of the A/D conversion of Complex I in the process of MPT pore formation and/or regulation during the reversible metabolic depression and strengthen the physiological relevance of the phenomenon. The verification of the proposal and a thorough examination of signaling pathways participating in the reversible metabolic depression in the lamprey liver are the aim of our future investigations.

## Abbreviations

LLM: Lamprey liver mitochondria
MPT: Mitochondrial permeability transition
EGTA: [Ethylenebis (oxyethylene-nitrilo)]tetraacetic acid
CsA: Cyclosporine A
RLM: Rat liver mitochondria
mtETC: Mitochondrial electron transport chain
P_i_: Inorganic phosphate
DTT: Dithiothreitol
BSA: Bovine serum albumin
FCCP: Carbonyl cyanide p-trifluoromethoxyphenylhydrazone
CCCP: Carbonyl cyanide 3-chlorophenylhydrazone
TMPD: Tetramethyl-p-phenylenediamine
Val: Valinomycin
DNP: 2, 4-Dinitrophenol
Nup: Nupercaine (dibucaine)
RR: Ruthenium Red
RCR: Respiratory control ratio
ΔΨ_mito_: Mitochondrial membrane potential
PN: Pyridine nucleotides
ROS: Reactive oxygen species
DB: 2, 3-Dimethoxy-5-methyl-6-n-decyl-1,4-benzoquinone
DCPIP: 2, 6-Dichlorphenolindophenol
TTFA: 2-Thenoytrifluoroacetone
FA: Fatty acids
Succ: Succinate
Py: Pyruvate
